# The 4R’s Framework of Nutritional Strategies for Post-Exercise Recovery: A Review with Emphasis on New Generation of Carbohydrates

**DOI:** 10.3390/ijerph18010103

**Published:** 2020-12-25

**Authors:** Diego A. Bonilla, Alexandra Pérez-Idárraga, Adrián Odriozola-Martínez, Richard B. Kreider

**Affiliations:** 1 Research Division, DBSS International SAS, Bogotá 110861, Colombia; dabonilla@dbss.pro; 2 Research Group in Biochemistry and Molecular Biology, Universidad Distrital Francisco José de Caldas, Bogotá 110311, Colombia; 3 Research Group in Physical Activity, Sports and Health Sciences (GICAFS), Universidad de Córdoba, Montería 230002, Colombia; 4 kDNA Genomics, Joxe Mari Korta Research Center, University of the Basque Country UPV/EHU, 20018 Donostia-San Sebastián, Spain; adrianodriozola@gmail.com; 5 Move Nutrition, Medellín 050021, Colombia; 6 School of Medicine, Universidad de Antioquia, Medellín 050010, Colombia; 7 Sport Genomics Research Group, Department of Genetics, Physical Anthropology and Animal Physiology, Faculty of Science and Technology, University of the Basque Country (UPV/EHU), 48940 Leioa, Spain; 8 Phymo Lab, Physiology and Molecular Laboratory, 08028 Barcelona, Spain; 9 Exercise & Sport Nutrition Laboratory, Human Clinical Research Facility, Texas A&M University, College Station, TX 77843, USA; rbkreider@tamu.edu

**Keywords:** physiological adaptation, rehydration, carbohydrate conformation, protein synthesis, muscle soreness, sleep hygiene

## Abstract

Post-exercise recovery is a broad term that refers to the restoration of training capacity. After training or competition, there is fatigue accumulation and a reduction in sports performance. In the hours and days following training, the body recovers and performance is expected to return to normal or improve. ScienceDirect, PubMed/MEDLINE, and Google Scholar databases were reviewed to identify studies and position declarations examining the relationship between nutrition and sports recovery. As an evidence-based framework, a 4R’s approach to optimizing post-exercise recovery was identified: (i) Rehydration—a fundamental process that will depend on the athlete, environment and sports event; (ii) Refuel—the consumption of carbohydrates is not only important to replenish the glycogen reserves but also to contribute to the energy requirements for the immune system and tissue reparation. Several bioengineered carbohydrates were discussed but further research is needed; (iii) Repair—post-exercise ingestion of high-quality protein and creatine monohydrate benefit the tissue growth and repair; and (iv) Rest—pre-sleep nutrition has a restorative effect that facilitates the recovery of the musculoskeletal, endocrine, immune, and nervous systems. Nutritional consultancy based on the 4R’s is important for the wise stewardship of the hydration, feeding, and supplementation strategies to achieve a timely recovery.

## 1. Introduction

Physical exercise-induced adaptations take place as a result of acute-to-chronic changes at the metabolic, cellular, tissue, and system level [1,2,3,4]. During physical exercise, several molecular modifications are involved within the muscle cell processes of ATP synthesis and degradation [5], to the point that the relationship between energy production and consumption (myocellular ATP/ADP ratio) represents a key point in the occurrence of muscle fatigue, which is characterized by an acute reduction in force and power in response to contractile activity [6]. Here, the significant increase in the intramuscular concentration of inorganic phosphate (Pi) and hydrogen ions (H^+^) is strongly correlated with the onset of neuromuscular fatigue [7].

**Figure 1:**
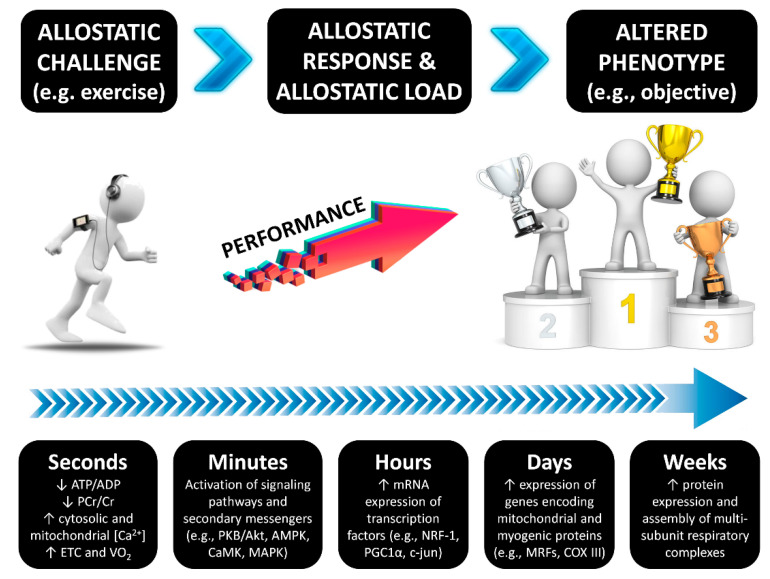
The time course of exercise-induced adaptations. Transient changes in metabolite sensing and signaling during/after exercise drive to gene transcription of early genes, myogenic regulators, genes of carbohydrate metabolism, lipid mobilization, transport and oxidation, mitochondrial metabolism and oxidative phosphorylation, and transcriptional regulators of gene expression and mitochondrial biogenesis [13]. DNA methylation is a regulatory point for transcription and may have a certain influence, although the current evidence suggest that exercise adaptations are regulated to a greater extent at the post-transcriptional level [14]. The time course changes following exercise have been described previously [15,16].

Considering the allostasis model, which proposes that efficient regulation of a biological system requires anticipating needs and preparing to satisfy them before they arise [8], nutritional strategies after exercise helps to refuel energy sources (e.g., muscle and liver glycogen), replace fluid and electrolytes, synthesize new proteins to counteract both catabolic state and exercise-induced damage, and improve immune system response [9,10,11]. All these aspects have a tremendous influence on the allostatic response and the allostatic load, which must be sustained for an appropriate interval of time. According to Edes and Crews (2017), “Allostatic load results from altered physiology across multiple systems secondary to stressors and related responses and, therefore, may be estimated using multisystem biomarker composites representing, for example, the neuroendocrine, metabolic, cardiovascular, and immune systems” [12]. For example, during post-exercise molecular mechanisms that regulate the adaptation to exercise training, there is a time-course alteration in the protein content and enzyme activities as the result of the down-stream activation or inhibition of certain signaling pathways that regulate gene expression (transcription and translation processes) [13]. In the end, these mechanisms will result in an altered phenotype that is in line with the athlete’s objectives, such as optimization in strength, cardiorespiratory fitness, power, agility, speed, exercise economy (VO_2_ at submaximal velocity), body composition (i.e., muscle hypertrophy), among other aspects of sports performance (Figure 1).

In this regard, the musculoskeletal tissue (as well as other tissues) possesses remarkable plasticity in response to repeated stimuli, such as exercise training, which results in phenotypic changes as an increase in the ability to sustain contraction and resist fatigue [14]. Therefore, to structure or periodize the recovery process, it is necessary to prepare the body physically and mentally to respond to the demands of training and competences, seeking not to interfere in the adaptation processes required to improve progressively [17]. Thus, recovery strategies will depend to a large extent on the proximity of the next session, the degree of physiological stress and the relevance of the next event. This determines how to rehydrate, replenish energy and consume the nutrients needed to improve tissue repair. This review article aims to summarize the nutritional strategies to optimize post-exercise recovery with emphasis on new carbohydrates forms.

## 2. Methods

A literature search using several databases (ScienceDirect, PubMed/MEDLINE, and Google Scholar) was conducted to identify studies and position declarations examining the relationship between nutrition strategies, dietary supplements and post-exercise recovery. The search string for all databases was “nutrition AND (exercise recovery OR sports recovery)” although further papers were sought by hand-searching. We prioritized papers written in English and published from 2010 onwards although selected publications before this cut-off were included as the basic body of evidence in the field. On the other hand, we excluded articles that did not analyze the effects on post-exercise recovery, abstracts or articles with no full-text version available, and studies focused on the effects of performance and image enhancing drugs. To emphasize in the description and role of new generation carbohydrates, the identification of additional studies was enriched by performing a hand-search with free language terms related to the effects of the administration of bioengineered carbohydrates on sports recovery and performance.

## 3. Results and Discussion

**Figure 2:**
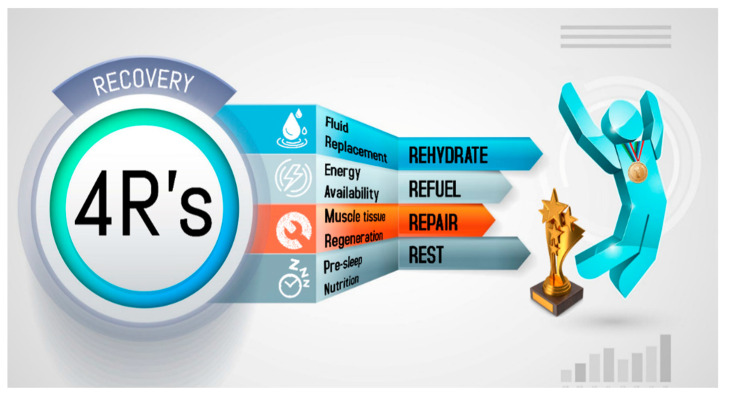
The 4R’s framework of nutritional strategies to optimize post-exercise recovery in athletes.

The execution of the search algorithm with Boolean operators resulted in 1816 references; nonetheless, we performed a screening of articles based on article type, text availability and suitability with the topic to discuss the following sections. To improve comprehension regarding the nutritional strategies that impact post-exercise recovery, a mnemonic entitled the 4R’s (Rehydrate, Refuel, Repair, and Rest) is introduced. This approach divides the nutrition intervention into four interrelated scenarios that follow the post-exercise time course in order to optimize the exercise-induced adaptations and recovery (Figure 2).

### 3.1. Rehydrate

One of the first goals during recovery is to replace any fluid and electrolyte deficits. Most physically active individuals sweat from 0.3 to 2.4 L·h^−1^, which depends on exercise intensity, duration, and environmental conditions such as altitude, heat, and humidity [18,19]. Moreover, individual characteristics (i.e., body mass, genetic predisposition, heat acclimatization state, physical fitness, and metabolic efficiency) might influence sweat rates for a given activity [20]. For instance, the highest sweat rate was registered at 3.7 L·h^−1^ for a world-class ultramarathon runner [21]. Thus, measuring pre and post-exercise body mass is a recommended practice to assess fluid status.

Rehydration is important, especially in team, endurance or ultra-endurance sports, where in many cases it is not possible to compensate for the loss of fluids and electrolytes that occur during exercise, particularly in hot and humid environments. As a general advise, for quick rehydration, it is recommended the consumption of 150% of the weight lost after exercise over a short recovery period (less than 4 h) [22,23], with a sodium concentration between 20 and 30 mEq·L^−1^ [18]. Athletes and practitioners should replenish three cups of fluid per pound of weight lost (~1.5 L·kg^−1^) and to make sure body mass is back up before the next training session. Furthermore, it has even been shown that consuming a sodium-containing drink between 40 and 60 mEq·L^−1^ can improve fluid retention and rehydration when there is little time between sessions or when there is moderate dehydration [24].

Rehydration can last between four and 24 h [9]. If recovery time and opportunities allow it, the consumption of sodium-rich foods, such as crackers, peanuts, bread, milk, cheese, ham, kabanos, and soups, may be sufficient to regain the state of euhydration. However, if the recovery time is less than 12 h, more aggressive rehydration strategies and the use of moisturizing beverages (e.g., glycerol) are required before the next training or competition [25]. One practical application to improve both the rate of rehydration and total fluid retention following exercise is the ingestion of glycerol [22,26]; however, professional advice is recommended to avoid potential gastrointestinal discomfort with any hyperhydration agent.

In the rehydration process, it has been found that neither the addition of potassium to the drink [27] nor the way of distributing the volume [28] nor the temperature influence the percentage of liquid preserved to be subsequently used by the body for rehydration [29]; however, it is believed that the delay in early rehydration after exercise is attributed to a reduction in sensations associated with thirst, and it is known that the taste and cool temperature of beverages can positively affect these sensations [30]. On the other hand, some drinks that can be used for rehydration and in turn help recovery has been successfully studied, such as chocolate milk [31,32,33].

Rehydration is a fundamental step in recovery, but what, how, when and how much will depend on the athlete and the particular event. We adhere to the position statement of the National Athletic Trainers’ Association to emphasize that education strategies for athletes should address personal sweat rates, hydration cues, and rehydration strategies that avoid both hypohydration and fluid overload [23].

### 3.2. Refuel

At the end of the exercise, there are several strategies to maximize muscle and liver glycogen replenishment, especially when two or more sessions are performed on the same day or when competing on consecutive days. For planning, it is necessary to consider the state of training, schedules, and the magnitude of the depletion of reserves, besides the type of exercise [34]. In this sense, the amount of carbohydrates is determined by the need to replenish muscle glycogen stores, and according to Jeukendrup (2017) [17], this is closely related to:

- Time to next training session or competition,
- Nutrition periodization to achieve adaptations,
- Need for muscle repair and growth,
- The amount consumed before and after as part of global requirements.

Although certain general recommendations can be given, the carbohydrate intake must be fine-tuned based on individual features, total energy daily expenditure, exercise training requirements, and the respective feedback from training performance in daily recovery [35]. In athletes with high body mass (e.g., basketball and rugby) or players under a weight loss program it might be better to reduce the energy intake to the needs of the previous category [36]. Additionally, resistance/power athletes do not need much carbohydrates as endurance athletes to maintain optimal liver and muscle glycogen; therefore, based on the exercise and sports nutrition review update of the International Society of Sports Nutrition [37], daily carbohydrate needs might be ranked as follows:

- Moderate duration/low-intensity training (e.g., 2–3 h per day of intense exercise performed 5–6 times per week): 5–8 g·kg^−1^ body mass·day^−1^
- Moderate to heavy endurance training (e.g., 3–6 h per day of intense training in 1–2 daily workouts for 5–6 days per week): 8–10 g·kg^−1^ body mass·day^−1^
- Extreme exercise programs or competition (+6 h per day or high competition frequency during the week): 10–12 + g·kg^−1^ body mass·day^−1^

Moreover, in the post-exercise period, it takes about four hours for carbohydrates to be digested and absorbed into muscle and liver tissues to be incorporated as glycogen. Hence, if rapid recovery is required due to a limited time period available, the priority should be to consume large amounts of daily carbohydrates (>8 g·kg^−1^ body mass·day^−1^) and to eat a high carbohydrate meal within two hours following exercise with at least 1.2 g·kg^−1^·h^−1^ for the first four hours of recovery [38]. Ingestion of a glucose polymer or the combination of glucose and fructose (sucrose) results in a fast replenishment of muscle glycogen stores whilst also minimizing gastrointestinal distress [39], and there is no need for protein and/or amino acid ingestion in order to enhance the insulin levels if sufficient carbohydrates are consumed (1.2 g·kg^−1^·h^−1^). In fact, higher insulin concentrations do not further increase the rate of muscle glycogen synthesis when carbohydrate intake is sufficient [34]. Slightly less carbohydrate plus protein (e.g., 1 g carbohydrate·kg^−1^ and 0.5 g protein·kg^−1^) within 30 min after exercise or carbohydrates along with caffeine may also be used to aid rapid glycogen resynthesis [37]. Additionally, compared to carbohydrate ingestion alone, multiday supplementation with creatine monohydrate along with an adequate amount of carbohydrates has been reported to have a higher positive impact on muscle glycogen synthesis [40].

The consumption of carbohydrates is not only important to replenish the reserves, but also to contribute to cover the energy requirements that are fundamental to help the competition of the immune system and the repair of the tissues [35]. Sports nutritionists, coaches and athletes should be cautious with the potential physiological implications of the relative energy deficiency in sport, which includes impaired metabolic rate, hormonal disruptions, menstrual dysfunction, reduced bone health, immunity, protein synthesis, and cardiovascular health [41,42].

Considering the potential impact of the type of carbohydrates on performance and recovery, some bioengineered formulations with different physicochemical characteristics are described in the next lines. These bioengineering processes refer to the application of theoretical and experimental methods of the basic sciences to produce new scientific knowledge with practical applications, such as the modification of the chemical structure of a molecule by means of biotechnological procedures that use microorganisms (e.g., bacteria) in order to produce substances with different metabolic responses since they are not found regularly in food. We recommend the reader to visit the Carbohydrate Structure Database (CSDB, http://csdb.glycoscience.ru) [43] and the database of Chemical Entities of Biological Interest (ChEBI) [44] to know in depth about advances, structures and different applications of the new generation of carbohydrates produced through different processes and that are of biological interest.

#### 3.2.1. Isomaltulose (BCSDB ID: 111249; ChEBI 18394)

Commercially known as Palatinose ^TM^, 6-O-α-D-glucopyranosyl-D-fructose (or isomaltulose) is a white and sweet disaccharide constituted of glucose and fructose that originates from an enzymatic rearrangement of the α-(1→2) bond of sucrose until the formation of an α-(1→6) bond by microbial sucrose isomerases (i.e., EC 5.4.99.11) [45]. Thus, isomaltulose is part of a category of carbohydrates called non-starch slowly digestible carbohydrates, which have unique glycosidic bonds that are different from the better-known or naturally occurring molecules; therefore, they are digested and absorbed at a different rate resulting in a changed glycemic and insulinemic response [46]. Evidence suggests that the use of isomaltulose instead of conventional sucrose has certain metabolic advantages that might be useful for metabolic syndrome patients [47], besides being an interesting ingredient for the development of "tooth-friendly" confectionery [48]. For instance, positive metabolic effects have been reported in physically-active type-I diabetic patients given that the consumption of isomaltulose alongside rapid-acting insulin reduction improved blood glucose responses to exercise and produced a similar high-intensity run performance compared with dextrose [49].

At a sporting level, isomaltulose ingestion (750 mL, 10% *w*/*v*) maintained a more stable blood glucose concentration and increased fat oxidation during exercise, resulting in improved cycling performance compared to maltodextrin consumption in endurance-trained athletes (VO_2max_ > 55 mL·kg^−1^·min^−1^) [50]. According to the authors, these results could be explained by the slower availability and low glycemic properties of isomaltulose, which allowed a greater reliance on lipid metabolism and might spare glycogen utilization during endurance exercise training. Similar results on fat oxidation during incremental exercise on cycle ergometer have been found recently in Japanese athletic population (long distance runners and triathletes) after isomaltulose ingestion (500 mL, 8%) [51]. In addition, after a soccer match simulation between male college soccer players, Stevenson et al. (2017) reported that the consumption of an 8% isomaltulose drink (0.36 mL kg^−1^ body mass at warm-up and 0.48 g kg^−1^ body mass at half-time) attenuated the decline in blood glucose concentration at 60 min in comparison to maltodextrin (−4% versus −19%, *p*  =  0.015). Moreover, both isomaltulose and maltodextrin lowered the rise in plasma epinephrine concentrations in response to prolonged exercise, albeit isomaltulose proved most effective at 90 and 120 min. Thus, the ingestion of isomaltulose beverages (8–10% *w*/*v*) may serve as an additional nutritional strategy for team sports players (e.g., soccer) when limited in-play feeding opportunities exist during prolonged high-intensity intermittent exercise [52]. Interestingly, during a heavy resistance training program, the recovery benefits of whey protein might be enhanced with the addition of β-Hydroxy-β-methylbutyrate (HMB) and isomaltulose, which has shown to have significant reductions in markers of muscle damage while improving athletic performance [53]. In addition, Amano et al. (2019) demonstrated that the ingestion of a 6.5% *w*/*v* isomaltulose drink plus electrolytes (Na^+^, Ca^2+^, Mg^2+^ and K^+^) may optimize post-exercise rehydration without affecting heat loss responses in healthy and physically active young men [54]. Therefore, current evidence suggests that low glycemic and low insulinemic properties of non-starch slowly digestible carbohydrates appear of a particular interest in sports nutrition and cognitive performance [55,56]. Further research is still needed due to the heterogeneity of the studies, and reports of negligible effects when conditions of the athletes are matched for carbohydrates and energy [18].

#### 3.2.2. Trehalulose (BCSDB ID: 111199; ChEBI: 79284)

**Figure 3:**
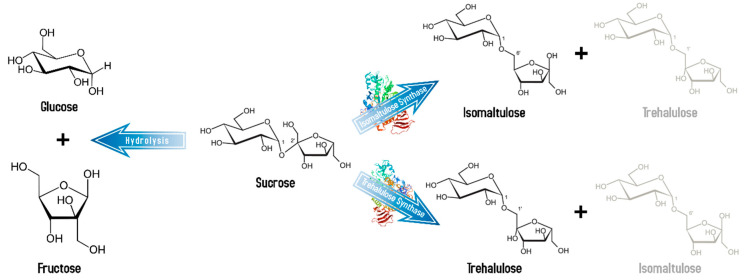
Isomaltulose and trehalulose production. Synthesis of the non-starch slowly digestible sucrose isomers by the isomaltulose synthase (PDB ID: 1M53) and trehalulose synthase (PDB ID: 1ZJA). Minor products of the reaction are represented by faint text. Blood glucose and insulin levels in humans after oral administration of these carbohydrates rise slower and reach lower maxima than after sucrose administration [60]. After hydrolysis of these disaccharides by the human small intestinal mucosal enzymes, fructose and glucose are metabolized as typical for these monosaccharides.

Chemically called 1-O-α-D-glucopyranosyl-D-fructose, trehalulose is a less known non-starch slowly digestible carbohydrate. This disaccharide is also an isomer of sucrose and is found in small amounts in honey but unlike isomaltulose it has no crystallization structure and is therefore found as an amorphous solid [57]. Trehalulose is synthesized by both the trehalulose synthase and as a coproduct by the same isomerase enzyme that synthesizes isomaltulose, but in generally smaller yields (Figure 3). Whereas the production of isomaltulose is kinetically favored, trehalulose is thermodynamically favored, so it tends to accumulate only after long reaction times [58]. Its relative sweetness to sucrose is between 0.4 and 0.7, which is particularly interesting for physically active diabetic individuals, or sportsmen, because of its similar properties to isomaltulose [59]; however, research is needed in this regard.

#### 3.2.3. Modified Starches and High-Molecular Weight Carbohydrates

Starch is the most common carbohydrate and can be classified as rapidly digestible, slowly digestible and resistant starch, where the speed parameter refers to how easily the enzymes hydrolyze the starch and, therefore, how quickly the blood glucose concentration is affected [61]. Starch modification has been carried out for a long time ago (approximately since the 1800s). Although several advances have been performed in regards to the chemical composition, which results in new nutritional properties, it is important to consider the consumer’s health and the environmental impact [62]. Generally, these modifications lead to carbohydrates with a higher number of ramifications, which increases the molecular weight of the compound. The high-molecular weight carbohydrates have the highest values among the available carbohydrates, ranging from 500,000 g·mol^−1^ to more than several million g·mol^−1^, in comparison to amylose molecules (between 150,000 and 1,000,000 g·mol^−1^), the conventional sports drink mixes (≈500 g·mol^−1^) or the mere sucrose (180 g·mol^−1^). This high molecular weight provides a very low osmolarity and therefore accelerates its passage through the stomach to the intestine [63]. Piehl-Aulin et al. [64] found, in well-trained male physical education students, that the osmolality of a carbohydrate drink influenced the rate of muscle glycogen resynthesis after its depletion by exercise when compared the effects of a polyglucoside with a molecular mass between 500,000 and 700,000 g·mol^−1^ (84 mOsm·L^−1^) and a carbohydrate drink with glucose monomers and oligomers with a molecular mass of approximately 500 g·mol^−1^ (350 mOsm·L^−1^). Hence, the consumption of high-molecular weight carbohydrates was proposed as an effective alternative to favor the post-exercise muscle (and probably hepatic) glycogen resynthesis, which might be of practical importance for athletes wishing to optimize performance during recovery following prolonged sub-maximal exercise [65]. Oliver et al. (2016) showed that this type of carbohydrate improved performance during subsequent repeated maximal explosive resistance training in strength-trained individuals [66]. Nonetheless, more studies are required under standardized conditions to have definitive conclusions according to the athletic population due to certain heterogeneity in the reported results [67,68,69].

#### 3.2.4. Cyclodextrins (ChEBI: 495055) and Derivates

The cyclodextrins are molecules consisting of α-D-glucopyranose units linked by α-(1→4) glycosidic bonds that resemble in shape a basket and are products derived from enzymatic digestion of starch using cyclodextrin glycosyltransferases (CGTases) obtained from microorganisms such as *Bacillus macerans*, *Klebsiella oxytoca*, *Bacillus circulans,* and *Alkalophylic bacillus* [70]. These CGTases act on pre-hydrolyzed starch through several transglycosylations to obtain some helical amylose molecules, which are finally cleaved at regular intervals of six, seven and eight glucose units in the absence of water to generate α-, β- and γ-cyclodextrin, respectively [71]. Although these three are the most abundant naturally occurring forms, besides the major products of CGTases, it has been reported that minor products contain trace amounts of larger cyclic glucans (δ-, ε-, ζ-, η-, and θ-cyclodextrins) [72]. Taking into account the stabilized and symmetrical form of cyclodextrins [73], these molecules have been used as drug delivery agents due to their ability to protect molecules from physical, chemical, and biological (enzymatic) degradation [74] and also their ability to solubilize hydrophobic drugs (also known as cyclodextrin complexation) [75].

Cyclodextrins have glycosidic bonds that salivary amylases are able to hydrolyze but they act slowly due to thermodynamic parameters [76]; therefore, cyclodextrins have very low glycemic indexes and provide a longer time of glucose supply to the bloodstream [77,78]. Almost 98% of these glucose-based rings pass through the small intestine without being absorbed, reaching the large intestine where they are metabolized by the intestinal microbiota to compounds such as acyclic maltodextrin, maltose, glucose, etc., and are finally absorbed [79]. As they are considered tasteless, odorless, non-digestible, non-caloric, and non-cariogenic saccharides, cyclodextrins represent dietary fibers useful in controlling body mass and blood lipid profile besides improving the intestinal microbiota through the selective proliferation of *Bifidobacterium*. These anti-obesity and anti-diabetic effects make them bioactive nutritional supplements and nutraceuticals [79].

**Figure 4:**
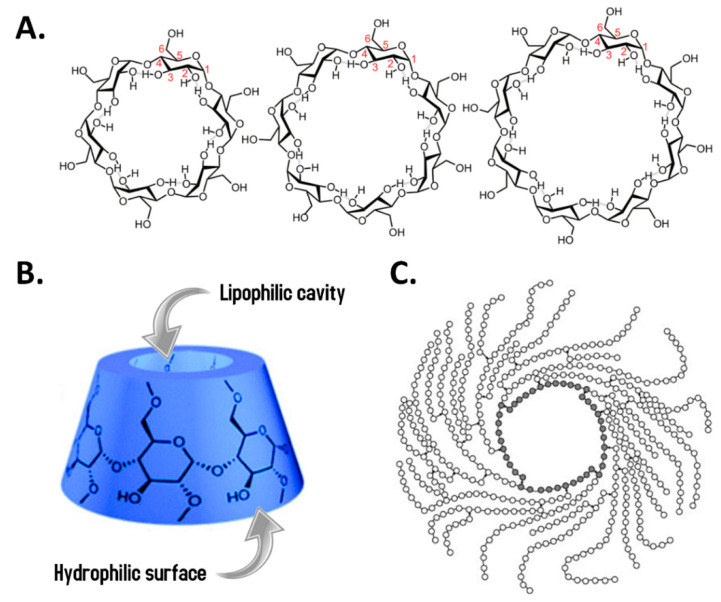
Molecular structures of common cyclodextrins and schematic representation of highly branched cyclic dextrin (HBCD). (**A**). Chemical structure of the α-, β- and γ-cyclodextrins, which contain 6, 7 or 8 glucose units, respectively. Image taken and modified from [86]. (**B**). The conical shape representation of cyclodextrins. These molecules have a truncated cone shape with a hydrophilic external surface (due to the hydroxyl groups on the C2, C3 and C6 atoms) and a hydrophobic cavity (due to the inner hydrogens (H3 and H5) pointing inward) [87]. Cyclodextrins have different water solubility: α- and γ-cyclodextrins have a relatively high solubility (145 and 232 g·L^−1^), whereas the β-type is much less soluble in water (18.5 g·L^−1^). Image taken and modified from [88]. (**C**). The schematic representation of HBCD is shown. Open circles represent glucose units while grey-filled circles are those conforming the main centered ring. Lines and arrows are α-(1→4) and α-(1→6) glycosidic bonds, respectively. Image taken from [89].

On the other hand, derivatives have been developed with potential application in the sports field such as highly branched cyclic dextrin (HBCD), which is a polymer of +900 glucose residues with a molecular mass between 160,000 and 400,000 g·mol^−1^. Due to its relatively high weight and narrow molecular distribution, HBCD has a low osmolarity (a 10% HBCD solution has an osmotic pressure of 9 mOsm·kg^−1^) that could favor gastric emptying, which is why it can be used to produce a sports drink without increasing the osmotic pressure too much, reaching 150 mOsm·kg^−1^ when added minerals, vitamins, and organic acids [80,81]. HBCD is produced from the processing of waxy corn starch using bacterial-derived α-amylase and α-(1→4)-glucan-branching enzyme (EC 2.4.1.18), a member of the broader group of glucanotransferases with validated results regarding the safety for human consumption [77]. Several studies have shown not only improvements at the gastrointestinal level [82] and a reduction on the gastric emptying time [78] but also positive effects on endurance sports performance [83], a decrease in the rating of perceived exertion during prolonged exercise [84] and even an attenuation in the stress hormonal response and reduction of urinary cytokine levels after extensive exercise in triathletes [85]. Figure 4 shows the molecular structures of common cyclodextrins and a schematic representation of HBCD.

These emerging modified carbohydrates not only offer metabolic and energy efficiency advantages (by regulating the use of substrates during exercise) but may also benefit certain species of microorganisms in the gut of athletes by acting as a source of non-digestible fiber [90]. In fact, the gut microbiome can rapidly respond to altered diet, potentially facilitating the diversity of human dietary lifestyles; for instance, the low consumption of dietary fiber and resistant starch may lead to a decrease in bowel movements resulting in decreased intestinal function, which would decrease the diversity of intestinal bacteria [91]. Thus, modulation of microbiota by means of probiotic/prebiotic supplementation has shown to ameliorate oxidative stress and inflammation and to improve performance in certain athletic populations but more rigorous studies are needed [92,93,94].

### 3.3. Repair

Scientific research has demonstrated that muscle protein synthesis (MPS) can be stimulated by either a physical allostatic challenge (e.g., resistance exercise stimulus) or by the ingestion of dietary protein, with synergistic responses when protein is consumed before and immediately after resistance exercise training [95]. According to the International Society of Sports Nutrition position stand on nutrient timing, post-exercise ingestion (immediately to 2 h) of high-quality protein food represents a robust stimulus that impacts positively on MPS; however, similar increases in MPS have been found when high-quality proteins are ingested immediately before exercise [96]. Indeed, taking into account the sport-specific daily needs, if an insufficient amount of protein is consumed, the athletes will develop and maintain a negative nitrogen balance, which is an indicator of protein catabolism and negatively affect recovery. Over time, this can lead to muscle wasting, injury, disease and intolerance to training [37]. Thus, the peri-exercise ingestion of insulinotropic protein and/or essential amino acid mixtures might stimulate post-exercise net muscle protein anabolism, and this might contribute to faster tissue growth and repair [34]. Similarly, recent findings have provided evidence that marathon runners that consume moderate amounts of protein post-exercise can have recovery benefits [97,98]. With a good grade of evidence, compared to ingestion of carbohydrate alone, co-ingestion of carbohydrate plus protein together during the recovery period have resulted in no difference in the rate of muscle glycogen synthesis but it improves net protein balance [18].

The International Society of Sports Nutrition position stand [95] about proteins for recovery is:

- Optimal dose of protein for athletes to enhance MPS are dependent upon age, energy intake (higher amount is needed under energy restriction), and recent resistance exercise stimuli. Post-exercise recommendations are 0.5 g of a high-quality protein per kilogram of body mass, or an absolute dose of 40 g. Protein per meal should be between 0.25 and 0.40 g of protein per kg of body mass, or absolute values of 20 g.
- Given the observed benefits of pre- and post-exercise protein ingestion, athletes’ tolerance should be assessed to determine the optimal time period during which to ingest protein. Notwithstanding, in spite of the anabolic effect of exercise is long-lasting (at least 24 h), athletes can take advantage of the higher muscle sensitivity to nutrient uptake after exercise due to the likely diminishment over time.

On the other hand, a large body of evidence suggests that creatine monohydrate supplementation (0.1 g·kg^−1^·day^−1^) not only optimize exercise adaptations and increase performance but also may reduce muscle damage and/or enhance recovery from intense exercise [99]. These effects are partially due to the optimization of the creatine kinase (CK) system which not only serves as a spatial/temporal buffer of ATP regeneration but also leads to positive regulation of anabolic signaling pathways (such as IGF-I and MAPK) and, hence, might promote faster tissue repair and recovery [100]. Additionally, it has been shown that chronic creatine supplementation prior to an exhaustive exercise bout and glycogen loading promotes greater glycogen resynthesis than just carbohydrate loading alone [101].

The role of antioxidants and anti-inflammatory substances are also highlighted in the post-exercise repair process. An increased antioxidant status leads to the reduction of oxidative stress caused by the production of reactive oxygen species during the inflammatory process [102]; therefore, the use of antioxidants can reduce muscle soreness and help with recovery in the short term, but high doses have also been linked to reduced training benefits in the long term. For example, Levers et al. (2015) found significant beneficial effects on serum markers of muscle catabolism, physiological stress, and inflammatory mechanisms after a short-term supplementation with 480 mg·day^−1^ of Montmorency powdered tart cherries surrounding a single bout of resistance exercise [103]. More recently, Brown et al. (2019) also reported that supplementation with Montmorency cherry concentrate can be considered as a practical nutritional intervention to reduce symptoms of muscle damage and improve post-exercise recovery on subsequent days in females [104]. It appears that secondary metabolites with antioxidant properties that are found in tart cherry extract might attenuate muscle soreness, strength decrement during recovery, and markers of muscle catabolism in resistance-trained individuals. In this sense, a systematic review and meta-analysis about the effects of antioxidants consumption showed that short-term polyphenol supplementation (e.g., quercetin) may boost athletic performance in predominately-trained males with an average intervention dose of 688 ± 478 mg·day^−1^ [105]. This has been reinforced by more recent research showing that acute and chronic supplementation with >1000 mg fruit-derived polyphenols per day will enhance recovery following muscle damage via antioxidant and anti-inflammatory mechanisms [106]; which suggest that fruit supplements could be used as part of the post-exercise recovery strategy although the need to educate and encourage athletes to consume more fruits and vegetables in the diet should be associated not only with recovery but also with health [107]. Similarly, curcumin is rich in polyphenols and has shown anti-inflammatory and antioxidant properties. The supplementation with 6 g of curcumin and 60 mg of piperine each day between 48 h before and 48 h after exercise-induced muscle damage have resulted in positive effects on recovery of the muscle function at 24 h and 48 h after the exercise [108].

The use of beetroot juice has been investigated in the management of hypertension [109,110], for the improvement of physical performance [111,112], and also in post-exercise recovery [113]. It has been found that acute supplementation of beetroot juice (250 mL) ×3 servings, two serving 24 h and 48 h following completion of 100-drop jumps attenuated muscle soreness and decrements in countermovement jump performance induced by eccentric exercise, while apparently having no effect on maximal isometric voluntary contractions, CK and some inflammatory markers (IL-6, TNF-alpha and IL-8) [114]. Interestingly, a betalain-rich concentrate of beetroots has shown to improve performance in competitive male and female triathletes and attenuated the increase of CK and fatigue suggesting an increase in recovery [115]. Other herbal and mushroom supplements that have promising effects to improve post-exercise recovery are *Zingiber officinale* [116], *Zingiber officinale* + *Bixa orellana L*. [117], *Rhodiola rosea* [118], *Cordyceps militaris* [119], and *Rhodiola rosea* + *Cordyceps sinensis* [120]. A root extract that warrants special attention is *Withania somnifera* (most known as ashwagandha) given some research have found ergogenic effects in athletic [121,122] and physically active individuals [123]. Moreover, the adaptogenic, anti-inflammatory, and antioxidant properties of ashwagandha [124,125,126] turns this herbal extract into a potential strategy to optimize recovery and promote exercise-induced adaptations. Although more research is required, dosages are between 300 and 500 mg of aqueous root extract twice per day.

On the other hand, it appears that supplementation with branched-chain amino acids (BCAA) after high-intensity exercise may favor a hormonal environment that contributes to attenuate the loss of strength, reduce muscle damage, and generate an anabolic environment [127]. Indeed, several systematic reviews and meta-analysis have concluded that BCAA supplementation (>200 mg·kg^−1^·day^−1^) may optimize recovery and mitigate muscle soreness following muscle-damaging exercise [128,129,130,131]. Notwithstanding, in resistance-trained males, the attenuation of muscular performance decrements and the observed decrease in plasma CK levels after BCAA supplementation is likely negligible when consumed with a diet consisting of ~1.2 g·kg^−1^·day^−1^ of protein [132]. The leucine-derived compound HMB has shown to improve work capacity recovery after high-intensity exercise [133], and may attenuate markers of muscle damage, augment acute immune and endocrine responses while preventing loss of lean body mass in catabolic situations [134]. A recent systematic review and meta-analysis by Rahimi et al. (2018) concluded that HMB may be seen as a recovery agent following exercise-induced muscle damage [135], but more research on recovery from injury that includes periods of extreme inactivity is needed given it does not consistently increase strength and/or lean mass or reduce markers of muscle damage [136]. Finally, it has been recently reviewed systematically if other foods, such as Pomegranate [137], cow’s milk [138], or Chocolate milk [139], might potentially improve exercise-performance and post-exercise recovery but further research is needed to extract definitive conclusions.

### 3.4. Rest

There is no doubt that sleep is an absolutely vital physiological function and one of the most important factors in post-exercise recovery [140]. It has been emphasized that naps, sleep extension, and sleep-hygiene practices seem to be advantageous to the performance by optimizing recovery [141]. In spite of the above, von Rosen et al. (2017) reported that the recommended amount of sleep during weekdays (8 h) was not obtained by 19% of 340 Swedish adolescent elite athletes of several disciplines during the autumn semester. Moreover, athletes sleeping more than eight hours and reached the recommended nutrition intake reduced the odds of suffering a new injury [142]. Portuguese elite female gymnasts have also found to have poor sleep habits with consequences on daytime sleepiness, sleep quality, and low energy availability associated with macro and micronutrients’ deficiencies [143]. In fact, according to a recent systematic review by Gupta et al. [144], athletes show a high overall prevalence of insomnia symptoms characterized by increased sleep latency, sleep fragmentation, non-restorative sleep, and excessive daytime fatigue. Currently, there is a lack of evidence and future research should focus on conducting sleep interventions among different athlete populations to address their specific sleep demands and disturbances [145].

It is known that eating the right combination of foods before going to sleep and what foods to avoid in the evening may be beneficial in enhancing sleep [146]. That is the rationale for the pre-sleep nutrition strategies, considering that several nutrients have been shown to improve sleep such as carbohydrates (high-glycemic index dinners), melatonin, tryptophan-rich protein, antioxidant-rich fruits (e.g., tart cherry juice and kiwi), and micronutrients [147]. Casein proteins, a type of secreted calcium (phosphate)-binding phosphoproteins, are among the most common nutrients used for pre-sleep nutrition given they are considered a high-quality protein source with high digestibility and bioavailability but with a slower digestion rate in comparison to whey [148]. Thus, the timing of nutrient intake is as important as the composition to fulfill the nutrition needs of the athletes [96]. Res et al. [149] reported for the first time that casein protein ingestion immediately before sleep was not only effectively digested and absorbed but also increased MPS and net protein balance in healthy young males that performed a resistance-training bout in the evening. Moreover, it has been demonstrated that pre-sleep casein protein ingestion augments the muscle adaptive response in terms of muscle mass and strength after a 12-week resistance exercise training program in young men in comparison to placebo [150]. Therefore, the extended window of opportunity as a result of the additive effects of resistance exercise training and protein ingestion on MPS makes the pre-sleep casein protein supplementation an effective nutrient timing strategy to optimize muscle conditioning and recovery [151] with no need to add extra leucine [152]. Although the positive effects of pre-sleep nutrition have been found particularly in resistance-type exercise training [153], more research is needed in endurance-trained athletes considering recent findings showed no improvement [154]. The available evidence and recommendations under this new paradigm of pre-sleep nutrition are:

- The consumption of 40–48 g of casein approximately 30 min before sleep improves post-exercise recovery and positively affect acute protein metabolism during an overnight period in healthy young adults [148,155].
- Ashwagandha supplementation (>150 mg aqueous root extract quaque hora somni) seems to be an effective nutritional strategy to improve sleep quality in healthy male and female subjects [156]; consequently, it should be also considered before sleep.

## 4. Conclusions

Several nutritional strategies may be used to optimize post-exercise recovery. The amount, composition, and timing for the consumption of fluids, electrolytes, macronutrients, antioxidants and/or supplements depend on the type of sport, the time between sessions, the level of preparation of the athlete, the convenience of the strategy, among other factors. There is not a single protocol to apply in post-exercise recovery. Based on the available evidence, we have identified a mnemonic entitled the 4R’s which stands for Rehydrate, Refuel, Repair, and Rest. These four R’s are not intending to replace the current methodologies or to establish a novel rigid paradigm in this regard, but to represent a strategic application of the nutritional strategies that should be taken into account during the recovery process. Taking into account the allostasis model, it is important to consider that each R represents a factor with tremendous influence on the allostatic response and the allostatic load that will impact the exercise-induced adaptions and recovery.

Rehydrate—It is necessary to guarantee the post-exercise consumption of at least 150% of the weight lost during the event (~1.5 L·kg^−1^) accompanied by sodium (if a faster replacement is required).

Refuel—The combined use of carbohydrates and proteins is a good strategy to replenish glycogen while contributing to tissue repair. Although new bioengineered formulations have been developed and introduced to the market, the source of macronutrients for men and women may be diverse and need not be limited to exclusively commercial sport nutrition products as long as an adequate amount of carbohydrates is provided at multiple intervals during post-exercise recovery [157]. Special attention should be paid to non-starch slowly digestible carbohydrates that have not been widely studied, such as trehalulose.

Repair—The ingestion of high-quality protein stimulates post-exercise net muscle protein anabolism and might contribute to faster tissue growth and repair. The use of certain supplements such as creatine monohydrate, tart cherry, beetroot juice, and possible ashwagandha might help to enhance recovery.

Rest—Optimal sleeping time and quality are necessary to benefit the allostatic response after exercise. Pre-sleep casein protein ingestion seems to be an effective strategy to boost the muscle adaptive response during a resistance exercise training program but more research is needed in endurance athletes.
